# Development of a Mechanism of Action-Reflective Cell-Based Reporter Gene Assay for Measuring Bioactivities of Therapeutic Glucagon-like Peptide-2 Analogues

**DOI:** 10.3390/molecules30091915

**Published:** 2025-04-25

**Authors:** Xiaoming Zhang, Chunyan Li, Zhe Deng, Chenggang Liang, Jing Li

**Affiliations:** 1 NHC Key Laboratory of Research on Quality and Standardization of Biotech Products, NMPA Key Laboratory for Quality Research and Evaluation of Biological Products, National Institutes for Food and Drug Control, Beijing 102629, China; zhangxm@nifdc.org.cn (X.Z.); liangchenggang@nifdc.org.cn (C.L.); 2 State Key Laboratory of Drug Regulatory Science, Beijing 102629, China; 3 Yunnan Institute for Food and Drug Control, Kunming 650106, China; lichunyan_ynmpa@yeah.net; 4 College of Life Science, Jilin University, Changchun 130012, China; zhe22@mails.jlu.edu.cn

**Keywords:** glucagon-like peptide-2 (GLP-2), teduglutide, reporter gene assay, bioactivity, method validation, RNA sequencing

## Abstract

Glucagon-like peptide-2 (GLP-2) is a gut hormone that plays a pivotal role in regulating intestinal epithelial cell growth and function, making it a promising therapeutic agent for intestinal damage and bone-related diseases. Nonetheless, the therapeutic potential of GLP-2 is substantially diminished due to its inactivation by dipeptidyl peptidase 4 (DPP-4). In recent years, advancements have been made in developing dipeptidyl peptidase 4 (DPP-4) resistant GLP-2 analogues with an extended half-life. The murine model with extensive experimental bowel resection maintained on parenteral nutrition has been used for assessing the physiology and pharmacology of GLP-2, and for the preclinical validation of GLP-2 analogues. However, it possesses certain limitations, such as complex procedure, considerable variability, and time-consuming nature. Consequently, there is a pressing need for the development of a cell-based bioassay to assess GLP-2 analogues. Here, we successfully developed a mechanism-of-action (MOA)-reflective cell-based reporter gene assay (RGA), utilizing a stable HEK293 cell line expressing the GLP-2 receptor and a luciferase reporter gene. This innovative approach allows for precise quantification of the potency of GLP-2 analogues. The RGA demonstrated good accuracy, linearity, precision, and specificity, with potential applications in stability testing, drug screening, and therapeutic monitoring of GLP-2 analogues. Moreover, RNA sequencing reveals the multi-target regulatory effect of GLP-2 analogues. The establishment of this RGA provides a valuable tool for evaluating the potency of GLP-2 analogues and the screening of potential therapeutic drugs targeting to GLP-2 receptor.

## 1. Introduction

Glucagon-like peptide-2 (GLP-2) is a 33 amino acid-long intestinal hormone that is encoded together with glucagon and glucagon-like peptide-1 (GLP-1), by the proglucagon gene. As a gastrointestinal hormone, GLP-2 is secreted postprandially by enteroendocrine L cells located throughout the intestinal tract [1]. Additionally, albeit in significantly smaller quantities, GLP-2 is also secreted from preproglucagonergic neurons situated in the brainstem and the hypothalamus [2]. GLP-2 is a pleiotropic hormone affecting multiple facets of intestinal physiology. Primarily, it regulates intestinal epithelial cell growth and functions that are crucial for digestion and absorption of nutrients, including enhancement of nutrient absorption, stimulation of blood flow, anti-inflammatory activity, improvement of barrier function, and modulation of motility [3,4]. Thus, GLP-2 emerges as a promising therapeutic target in intestinal damage. It was found that GLP-2 also plays an important role in bone turnover, thereby possessing potential for the treatment of bone-related diseases.

GLP-2 exerts its effects largely through interaction with the GLP-2 receptor (GLP-2R), which belongs to the class B1 of the G protein-coupled receptors. However, it is noteworthy that GLP-2R has been localized to central nervous system and several intestinal cell types that do not include the proliferating crypt cells [5]. This led to the hypothesis that GLP-2 acts indirectly through a complex network of indirect mediators that induce diverse signaling pathways, which remains poorly understood. For instance, GLP-2 has been shown to enhance the weight of small and large intestinal weight by stimulating epithelial cell proliferation and inhibition of apoptosis. It has been demonstrated that GLP-2 acts through an IGF-I-dependent pathway to activate intestinal crypt β-catenin, which integrates signals from multiple pathways to regulate the balance between crypt cell proliferation and differentiation [6]. The antiapoptotic response action of GLP-2 has been linked to the cAMP pathway involving both protein kinase A (PKA)-dependent and -independent mechanisms [7]. BHK fibroblasts stably transfected with the rat GLP-2R exhibit dose-dependent cAMP accumulation in response to GLP-2 administration [8,9].

The native form of GLP-2 undergoes rapid inactivation by the enzyme dipeptidyl peptidase 4 (DPP-4), which excises the two N-terminal amino acids, resulting in the truncated and inactive GLP-2 (3–33). This truncated form is subsequently degraded by endopeptidases and presumably cleared via renal excretion [10]. The inactivation of GLP-2 by DPP-4 significantly reduces its potential as a therapeutic agent. Consequently, DPP-4 resistant GLP-2 analogues with an extended half-life were developed [11,12]. Teduglutide (Gattex, Revestive) is the first and the only GLP-2 analogue that has been approved for medical treatment of short bowel syndrome (SBS). Teduglutide has an Ala-to-Gly substitution at position 2 to eliminate the DPP4 cleavage site ([Gly2] hGLP-2 (1–33)), and exhibits a terminal half-life of 2 to 3 h when administered subcutaneously to humans [13]. SBS is a severe and chronic disease characterized by malabsorption due to physical or functional loss of significant portions of the small intestine [14]. Managing SBS is challenging and requires careful monitoring to ensure nutritional and fluid needs are met through parenteral nutrition (PN). However, long-term PN can lead to serious complications. It has been found that native GLP-2 and teduglutide increase intestinal absorption by promoting intestinal growth and delays accelerated gastric emptying, gastrointestinal transit, and hypersecretions in SBS [15,16]. In patients with SBS, daily treatment with teduglutide led to a significant reduction in PN requirements and an improvement in intestinal function [14,17].

In addition to teduglutide, various additional analogues of GLP-2 with reduced clearance relative to GLP-2 are under development for SBS, including apraglutide, glepaglutide, and elsiglutide [18]. In a head-to-head preclinical study, apraglutide demonstrated a longer half-life and greater intestinotrophic effect in rat, compared to teduglutide and glepaglutide [2]. In Phase II clinical trials for patients with short bowel syndrome (SBS), apraglutide demonstrated a significant reduction in patients’ dependence on PN support, indicating its broad application prospects as a potential new therapy for rare gastrointestinal diseases [19,20]. Glepaglutide incorporates a number of design strategies presumably to optimize pharmacokinetic and potency properties, and is primarily used in research for inflammatory bowel disease and Crohn’s disease. Glepaglutide is also being developed as a potential treatment option for SBS [21]. Elsiglutide can increase cell proliferation and reduce intestinal cell apoptosis, showing great potential in the treatment of gastrointestinal mucositis, including chemotherapy-induced diarrhea [22].

While great excitement has been engendered by the clinical success of GLP-2 analogues, the development of appropriate in vitro bioassays for measuring potency and characterizing the biological properties of GLP-2 analogues has not been reported. The murine SBS model with extensive experimental bowel resection maintained on PN has been used for assessing the physiology and pharmacology of GLP-2, and for the preclinical validation of GLP-2 analogues [23,24,25]. However, the SBS model is not suitable for bioassay development, since it has certain limitations, such as complicated procedures, a time-consuming nature, high variability, and the sacrifice of the experimental animals after treatment. The reporter gene assay (RGA)—which is mechanism-of-action (MOA)-related, less variable, accurate, and precise—has been adopted as alternative assay for the bioactivity determination of biopharmaceuticals in quality control (QC) in recent years [26,27,28]. Here, we develop a novel RGA that engages GLP-2R and the subsequent signaling pathway. This cell-based bioassay is suitable for lot release and stability testing, as well as for biological property characterization of the GLP-2 analogues.

## 2. Results

### 2.1. Generation of the HEK293-GLP2R-Luc Cell Line

**Figure 1:**
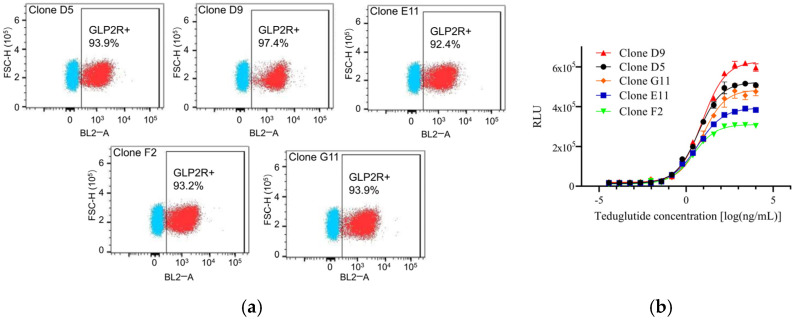
Generation of the HEK293-GLP-2R-Luc cell line. (**a**) Cytometry analysis of GLP-2R expression. (**b**) Identification of cell function with RGA.

**Figure A1:**
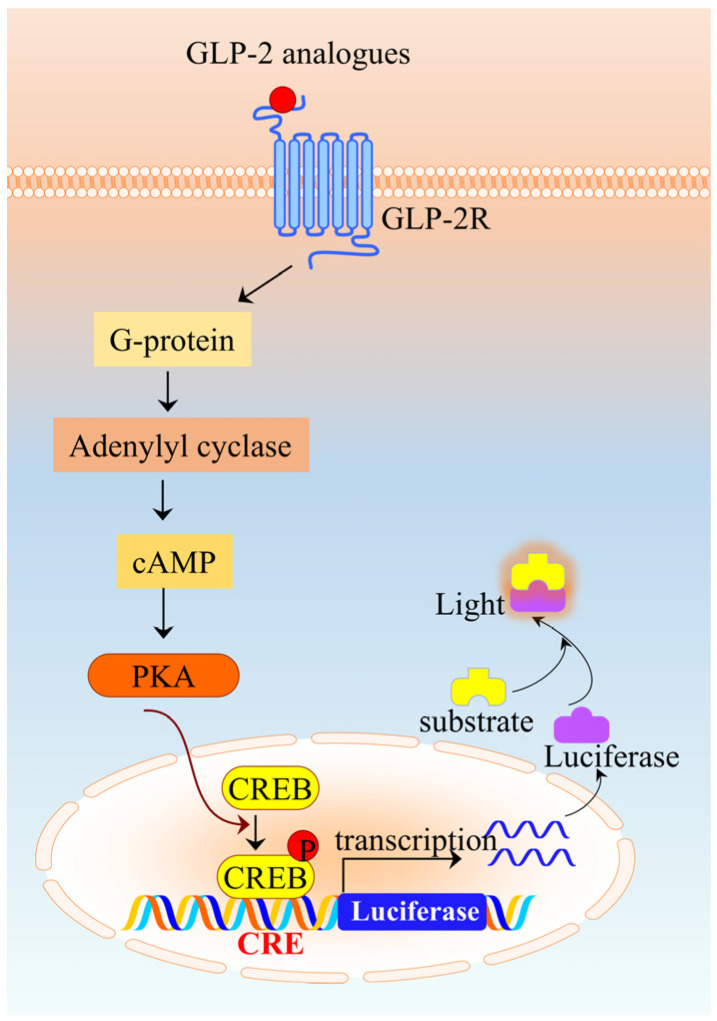
Schematic of the GLP2-PKA-CREB signaling pathway.

The intracellular effects of GLP-2 are initiated by binding to its cell membrane-spanning receptor, the GLP-2R. Following GLP-2 binding, the intracellular cAMP biosynthesis is elevated, leading to activation of PKA, followed by phosphorylation of the cAMP-response element binding protein (CREB), which then induces the transcription of several GLP-2 target genes. In order to develop a novel RGA for the determination of GLP-2 bioactivity, we constructed a HEK293-GLP-2R-Luc cell line based on the GLP-2R/PKA/CREB signaling pathway (Figure A1). This was achieved by stably expressing a luciferase reporter gene and GLP-2R in HEK293 cells. The transcription of the luciferase reporter gene is controlled by a cis-regulatory element (CRE) that can be recognized by CREB and activated by GLP-2. Specifically, we sequentially infected HEK293 cells with lentivirus particles carrying the luciferase gene under the control of CRE and lentivirus particles expressing GLP-2R. Resistant clones were selected in the presence of puromycin and zeocin, and then subcloned by limited dilution. The expression of GLP-2R was then confirmed with flow cytometry analysis. As shown in Figure 1a, five clones (D5, D9, E11, F2, G11) were subjected to cytometry analysis, and all clones showed ectopic expression of GLP-2R. The positive rates of GLP-2R for the monoclonal clones D5/D9/E11/F2/G11 were 93.9%, 97.4%, 92.4%, 93.2%, and 93.9%, respectively. Furthermore, all five clones showed high response to teduglutide treatment, and clone D9 was selected for subsequent experiments since it was more sensitive to GLP-2 (10 µg/mL for 6 h, EC50 of 4.6 ng/mL) and had the highest lower/upper asymptotes (~31.4) compared to the other 4 clones (Figure 1b). This clone was then designated as HEK293-GLP-2R-Luc cell line and employed to establish a RGA for GLP-2 analogues.

### 2.2. Reporter Gene Assay Optimization

**Figure 2:**
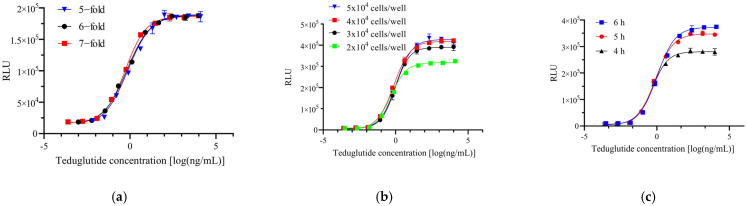
Reporter gene assay optimization. (**a**) Dilution ratio optimization. (**b**) Cell density optimization. (**c**) Incubation time optimization.

To determine the optimal RGA conditions, we optimized the RGA via the following parameters: dilution ratio, incubation time, and cell density. When one condition was optimized, the other conditions were held constant. To establish the most reliable sigmoidal dose–response curve, we systematically evaluated 5-, 6-, and 7-fold serial dilutions of teduglutide, starting from an initial concentration of 10 µg/mL. To determine the proper cell density, we investigated 4 different cell densities: 2 × 10^4^, 3 × 10^4^, 4 × 10^4^, and 5 × 10^4^ cells per well of 96-well white plate. Finally, 3 different incubation times (4, 5, and 6 h) were investigated. As shown in Figure 2a, the point distribution of the four-parameter logistic (4PL) curves obtained were all complete, but the 4PL curve obtained from a 7-fold dilution is the most uniform. An increased drug response was seen when the cell number increased from 2 × 10^4^ cells per well to 4 × 10^4^ cells per well, but no further increase in drug response was seen when the number of target cells was at 4 × 10^4^ cells/well (Figure 2b). A concentration-dependent drug response was seen after 4 h of incubation. A stronger response (higher upper asymptote) was observed when the incubation time was increased to 5 h and 6 h (Figure 2c). Thus, the optimized conditions for the subsequent experiments were as follows: (1) 7-fold dilution of teduglutide for 10 points; (2) 4 × 10^4^ cells per well for the optimal cell density; (3) the optimal incubation time was 5 to 6 h. The optimized experimental parameters were systematically applied to all GLP-2 analogs evaluated in this study, ensuring uniformity across all experimental cohorts.

### 2.3. Validation of the Reporter Gene Assay

In order to prove the relative accuracy, linearity, precision, and specificity of the RGA, we comprehensively validated the RGA according to the ICH Q2 (R2) guidelines and the Chinese Pharmacopoeia (ChP), as well as the United States Pharmacopoeia (USP).

**Figure 3:**
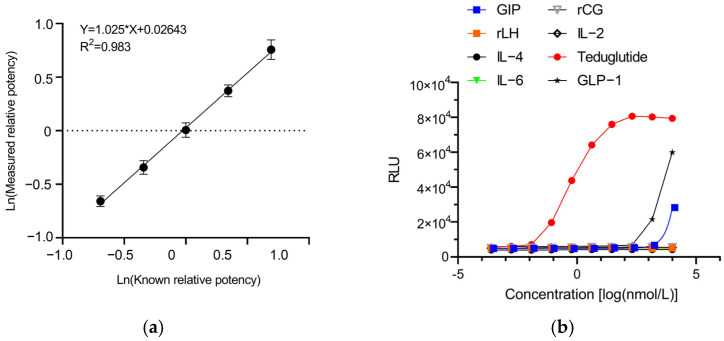
Validation of the reporter gene assay. (**a**) The correlation of expected and measured bioactivity between the range of 50% to 200%. (**b**) Validation of the specificity of the RGA.

Table 1 showed the average and 90% confidence interval (CI) of validation results in the Ln scale, as well as corresponding potency, relative bias, and the geometric coefficient of variation (%GCV). The RGA has acceptable relative bias at levels from 50% to 200%, yielding mean relative bias region of −0.034 to 6.60, which fall within the acceptance region of ±12% relative bias. The slope of the linear curve was 1.025, which falls within the acceptance region of 0.8 to 1.25 (Figure 3a). The relative bias and slope of the linear curve indicated good relative accuracy. The mean %GCV of five relative potency levels were between 4.97% and 9.46%, which were below 20%, indicating high intermediate precision of the novel RGA. Furthermore, the linear curve of Ln measured potency versus Ln target potency also showed excellent linearity with R^2^ of 0.983. Taken together, these results demonstrated the novel RGA had acceptable relative bias, intermediate precision, and linearity within the range of 50% to 200%.

Specificity describes the ability of this RGA to assess teduglutide exclusively. GLP-2R, glucose-dependent insulinotropic peptide receptor (GIPR), and GLP-1R all belong to the class B GPCR subfamily, which is mainly expressed in the gut, pancreas, and brain [29]. We thus investigated the responsiveness of HEK293-GLP-2R-Luc cells to GIP and GLP-1. As shown in Figure 3b, the concentration–response curve was unsuccessfully induced by GIP and GLP-1 that belongs to the same family as GLP-2. Furthermore, we randomly examined the response of HEK293-GLP-2R-Luc cells to two more hormones, rCG and rLH, the receptor of which is also a G-protein-coupled receptor [30]. Several cytokines, including IL-2, IL-4, and IL-6, were also used to validate the specificity of the RGA. As expected, no dose–dependent response curve was induced by rCG, rLH, IL-2, IL-4, and IL-6. Collectively, these results indicated that the established RGA is highly specific to teduglutide.

### 2.4. Stability Testing of Teduglutide with the RGA

**Figure 4:**
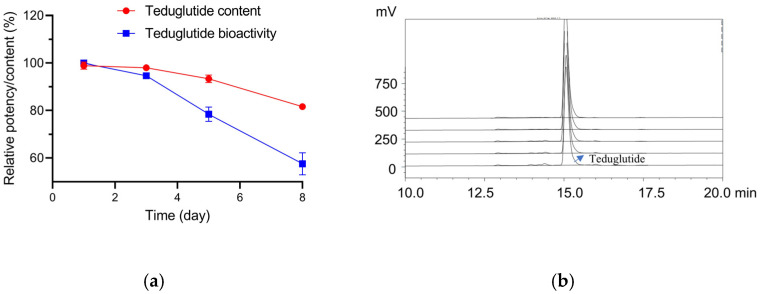
Stability testing of teduglutide with the RGA. (**a**) Bioactivity analysis and teduglutide content analysis with the RGA and HPLC, respectively. (**b**) HPLC chromatogram showing teduglutide main peak.

We expected that the new RGA could be applied to the biological stability study of teduglutide. According to ICH Q5C “Stability Testing of Biotechnological/Biological Products” and ChP General Rule 9402 “Stability Testing of Biological Products”, the stability test should explore and optimize the test conditions based on the product’s own characteristics. The temperature in the influence factor test should be high enough to observe the inactivation, denaturation, or degradation of the sample and exceed the quality standard limit. To this end, teduglutide drug substance (DS) was subjected to a temperature of 37 °C for durations of 1, 3, 5, 8, 12, and 14 days, respectively. To our surprise, the DS solution develops a clear colloidal precipitate after incubation for 12 or 14 days at 37 °C, rendering it unsuitable for bioactivity determination. As illustrated in Figure 4a, The relative potency of teduglutide, when compared to non-treated disease state (DS), progressively diminishes with increasing incubation time at 37 °C, as evidenced by a decline in EC50 values from 100.5% to 57.5%. The teduglutide contents were then determined using high-performance liquid chromatography (HPLC). As expected, the content of teduglutide decreased from 99.9% to 81.6% (Figure 4b), which suggested that the reduction of teduglutide bioactivity was successfully monitored via our new RGA.

### 2.5. Stability and Application of the New RGA

**Figure 5:**
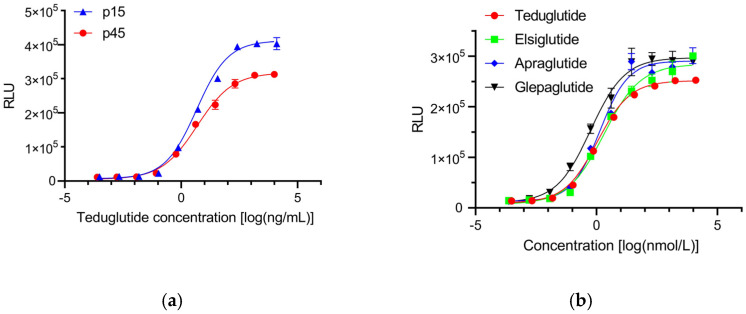
Stability and application of the new RGA. (**a**) Stability of HEK293-GLP-2R-Luc cells of different passages. (**b**) Bioactivity analysis of GLP-2 analogues with the new RGA.

The cell passage stability serves as an indicator of system stability, and plays a pivotal role in assessing the consistency of bioactivity determination. As thus, two different passages (p15 and p45) of HEK293-GLP-2R-Luc cells after cryopreservation and recovery were applied to measure the relative bioactivities of teduglutide reference sample. As shown in Figure 5a, two parallel teduglutide dose-dependent curves were obtained, with the lower/upper asymptotes decreased slightly from 32 in p15 cells to 28 in p45 cells, while the EC50 of both cells were similar (4.6 ng/mL in p15 cells, 4.8 ng/mL in p45 cells). We then measured the bioactivity of 3 batches of teduglutide DS and 3 batches of drug products (DP) using the two passage cells simultaneously. There were no statistical differences in the relative bioactivity of teduglutide between the two passages. Collectively, these results suggest that our HEK293-GLP-2R-Luc cells exhibited sufficient stability.

In order to explore the application of the new RGA in other GLP-2 analogues, apraglutide, glepaglutide, and elsiglutide were synthesized and applied to bioactivity investigation using the HEK293-GLP-2R-Luc cells (Table 2). All three GLP-2 analogues were diluted to 2.665 µmol/L, and further diluted 7-fold for 10 concentration points. As expected, all three analogues induced sigmoid curves similar to those induced by teduglutide (Figure 5b). These findings indicated that the bioactivity of GLP-2 analogues was successfully monitored via our new RGA.

### 2.6. The Multi-Target Regulatory Effect of GLP-2 Analogues Was Revealed by RNA Sequencing

GLP-2 regulates proliferative and cytoprotective pathways in the intestine. Administration of GLP-2 to rodents enhances the growth of the intestinal epithelium by stimulating the proliferation of crypt cells and inhibiting the apoptosis of enterocytes [8,31]. A study on GLP-2 biology indicates that the activation of GLP-2R triggers a cAMP- and PKA-dependent pathway, which is associated with the inhibition of apoptosis. Additionally, it couples with the activation of the Ras/MAPK pathway and stimulates DNA synthesis [32]. GLP-2 has also been reported to stimulate neuronal differentiation of enteric neurons and glial cells in culture via the PI3K/Akt/mTOR pathway [33].

**Figure 6:**
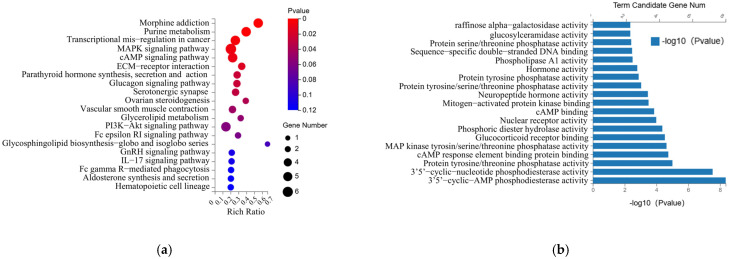

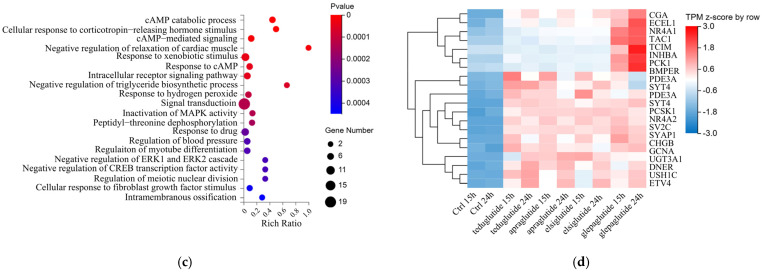
RNA sequencing analysis between teduglutide, apraglutide, glepaglutide, elsiglutide, and control groups. (**a**) Enriched KEGG bubble plots of differential metabolite pathways. (**b**) GO enrichment of molecular function (MF). (**c**) GO enrichment of biological process (BP). (**d**) Heatmap showing the top 20 significantly upregulated genes.

RNA sequencing was applied to elucidate whether GLP-2 analogues also activate proliferative and cytoprotective pathways in HEK293-GLP-2R-Luc cells. A total of 83 differentially expressed genes (DEGs) comprised of 76 upregulated genes and 7 downregulated genes with more than 2-fold change were obtained from teduglutide, apraglutide, glepaglutide, and elsiglutide-treated HEK293-GLP-2R-Luc cells in comparison to DMSO control-treated cells (Appendix A, Table A1). Notably, 370 DEGs, comprising 283 upregulated genes and 87 downregulated genes with a fold change greater than 2 are differentially expressed exclusively in HEK293-GLP-2R-Luc cells treated with glepaglutide (Table A2). The KEGG enrichment analysis of the DEGs listed in Table A1 revealed that these DEGs were predominantly enriched in pathways such as the MAPK signaling pathway, cAMP signaling pathway, glucagon signaling pathway, and PI3K-Akt signaling pathway (Figure 6a). GO enrichment of molecular function (MF) revealed enrichment in cAMP phosphodiesterase activity, protein tyrosine/threonine phosphatase activity, MAP kinase tyrosine/serine/threonine phosphatase activity, mitogen-activated protein kinase binding (Figure 6b). GO enrichment of biological process (BP) involved a wide range of physiological activities, including cAMP catabolic process, response to xenobiotic stimulus, response to hydrogen peroxide, regulation of blood pressure, negative regulation of ERK1 and ERK2 cascade, and cellular response to fibroblast growth factor stimulus (Figure 6c). The top 20 significantly upregulated genes in teduglutide, apraglutide, glepaglutide, and elsiglutide-treated HEK293-GLP-2R-Luc cells were listed in the heatmap (Figure 6d).

## 3. Discussion

The diverse functions of GLP-2 highlight its significance in maintaining intestinal health and physiology. As a naturally occurring gut hormone, GLP-2 not only promotes nutrient absorption by decreasing intestinal motility and permeability, but also plays a pivotal role in stimulating intestinal epithelial cell growth and function [34]. This multifaceted action of GLP-2 is crucial for the maintenance of the intestinal barrier, which is essential for preventing the translocation of harmful bacteria and toxins into the bloodstream [35]. The development of GLP-2 analogues, such as teduglutide, apraglutide, glepaglutide, and elsiglutide, has further expanded the therapeutic potential of GLP-2. These analogues exhibit extended half-lives and enhanced bioactivities, making them suitable for the treatment of various intestinal disorders [36].

In this study, we have established a robust and reproducible cell-based bioassay to measure the potency and characterize the biological activities of GLP-2 analogues. The RGA utilizes HEK293 cells stably expressing the GLP-2 receptor and a luciferase reporter gene, creating a sensitive and specific system for detecting GLP-2-mediated signaling. The RGA has been meticulously validated according to international guidelines, demonstrating its accuracy, linearity, precision, and specificity. These validation studies have confirmed that the new RGA is capable of measuring the bioactivity of teduglutide, as well as other GLP-2 analogues. Moreover, the new RGA can detect heat-stressed teduglutide sensitively, indicating its potential application in stability testing of GLP-2 analogues. The passage stability of the HEK293-GLP-2R-Luc cells was also assessed, ensuring the consistency and reliability of the bioactivity determinations over time.

RNA sequencing analysis identified a total of 83 differentially expressed genes in GLP-2 analogue-treated cells, providing valuable insights into the potential mechanisms of action of GLP-2 analogues. The upregulated genes are involved in a wide range of physiological activities, including cAMP catabolic process, response to xenobiotic stimulus, response to hydrogen peroxide, gluconeogenesis and lipogenesis regulation, and muscle growth and differentiation, which is consistent with previous reports [6,31,37]. This suggested that the HEK293-GLP-2R-Luc cells may potentially be useful as a cellular model for analysis of GLP-2R signaling and the screening of potential therapeutic drugs targeting to GLP-2R.

The GO enrichment analysis of the biological processes associated with these DEGs further supports the pleiotropic effects of GLP-2 analogues. For instance, the upregulation of genes involved in cAMP catabolic process and response to hydrogen peroxide indicates that GLP-2 analogues may have antioxidant and cytoprotective effects [38]. Similarly, the modulation of genes related to gluconeogenesis and lipogenesis regulation suggests that GLP-2 analogues may influence metabolic pathways and energy homeostasis [5,37]. It is worth noting that this study also found that GLP-2 analogues significantly regulated the expression of multiple genes, including NDRG1, NR4A2, etc. NDRG1 has been involved in cell growth and differentiation [39]. NR4A2 is important for the differentiation and maintenance of meso-diencephalic dopaminergic neurons during development [40]. Transcriptome data have revealed a rich GLP-2-related regulatory network, which provides a molecular-level explanation for the clinical observation that GLP-2 analogues improve intestinal barrier function. However, future research still needs to integrate multi-omics methods and establish intestinal-specific cell GLP-2 receptor knockout models, intestinal organoids, etc., to precisely dissect the molecular mechanisms of these effects. In addition, considering the potential application value of GLP-2 analogues in metabolic diseases such as obesity and diabetes, in-depth exploration of their role in energy metabolism will have significant translational medical significance.

It is of note that HEK293-GLP2R-Luc cells treated with glepaglutide exhibited a significantly higher number compared to the other three GLP-2 analogues-treated cells. This suggested that glepaglutide may have a distinct effect on gene expression within these cells. Additionally, the unique gene expression profile induced by glepaglutide may provide insights into its potential for treating specific gastrointestinal disorders or conditions where modulation of these pathways could be beneficial. Additional studies are needed to further elucidate the roles of these DEGs and their interactions in the context of glepaglutide-mediated cellular responses.

In conclusion, the establishment of this MOA-reflective cell-based bioassay represents a significant advancement in the evaluation of GLP-2 analogues. The new RGA is sensitive, specific, and reproducible, making it suitable for quality control, lot release, and stability testing, as well as for biological property characterization of the GLP-2 analogues. Furthermore, the RNA sequencing analysis has provided valuable insights into the potential mechanisms of action of GLP-2 analogues, paving the way for future research in this area. Overall, this study contributes to the ongoing efforts to develop novel therapies for intestinal damage and other related disorders.

## 4. Materials and Methods

### 4.1. Materials

The human embryonic kidney 293 (HEK293) cell line was purchased from ATCC (Manassas, VA, USA). HEK293-GLP-2R-Luc cells were cultured in Dulbecco’s modified eagle medium (DMEM) supplemented with 10% fetal bovine serum (FBS), 200 µg/mL zeocin, and 3 µg/mL puromycin. Both antibiotics, recombinant human Interleukin-2 (IL-2, PHC0027), IL-4 (200-04), IL-6 (200-06), and mouse IgG isotype control were purchased from Thermo Fisher Scientific (Waltham, MA, USA). Recombinant human chorionic gonadotrophin (rCG, 410021) was supplied by NIFDC (Beijing, China). Recombinant human luteinizing hormone (rLH) and teduglutide were provided by different manufacturers and stored at 4 °C or −80 °C in our laboratory. DMEM and FBS were purchased from Gibco (Grand Island, NY, USA). Britelite plus reporter Gene Assay System (6066769) was purchased from Revvity (Waltham, MA, USA). The pLU.cmv.Zeo and pLU.CMV.Luc.PGK.PURO were obtained from dlbiotech (Suzhou, China). The mouse anti-human GLP-2R monoclonal antibody (Clone # 413801) was purchased from R&D (Minneapolis, MN, USA).

### 4.2. Peptide Synthesis

GIP, GLP-1, glepaglutide, elsiglutide, and apraglutide peptides used in this study were synthesized by GenScript (Nanjing, China). All these peptides were prepared as trifluoroacetic acid salts by solid-phase peptide synthesis, subsequently purified by reverse-phase HPLC, and definitively verified via mass spectrometry. All the peptides had purity no less than 99% as measured by analytical HPLC. Amino acid sequences of the GLP-2 analogue peptides used in this study were identified from a published report [2].

### 4.3. Plasmid Constructions and Virus Production

To generate constructs for viral packaging, the CRE-miniP (TGACGTCAGCTGCCAGATCCCATGGCCGTCATACTGTGACGTCTTTCAGACACCCCATTGACGTCAATGGGAGAACAGATCTGGCCTCGGCGGCCAAGCTTAGACACTAGAGGGTATATAATGGAAGCTCGACTTCCAG) was synthesized and cloned into the pLU.CMV.Luc.PGK.PURO vector via the Cla I and Xba I sites. The full-length cDNA of human GLP-2R was synthesized, cloned, and inserted into the pLU.cmv.Zeo vector using BamH I and Sal I sites. Virus production was conducted as described previously [41]. Briefly, HEK293LT cells cultured in 60 mm dishes were co-transfected with 4 mg of lentiviral transfer constructs (pLU.CRE-miniP.Luc.PGK.PURO or pLU.cmv-GLP2R.Zeo), 2 mg of the packaging construct pCMV-DR8.91 (encoding HIV-1 gag-pol), and 0.4 mg of PMD2. G (encoding the VSV-G envelope) using the calcium phosphate method. Lentivirus-containing supernatants were harvested on two consecutive days and concentrated.

### 4.4. Generation of the HEK293-GLP-2R-Luc Cell Line

To generate a HEK293 cell line stably expressing CRE-driven luciferase and GLP2R, the CRE-miniP.Luc virus-containing cell supernatants were diluted in DMEM in the presence of 8 µg/mL polybrene before being added to HEK293 cells. To obtain a stable cell pool, the medium was replaced 48 h after incubation, with fresh culture medium containing 3 µg/mL puromycin. After about 3 weeks selection, the cell pool was then further infected with pLU.cmv-GLP2R.Zeo virus and selected with 200 µg/mL Zeocin and 3 µg/mL puromycin. After another 3 weeks selection, stable HEK293-GLP-2R-Luc monoclonal cell lines were obtained by limited dilution. The expression of GLP-2R was examined using flow cytometry. After that, the positive clones were routinely maintained in selective medium.

### 4.5. Flow Cytometry Analysis

HEK293-GLP-2R-Luc cells were rinsed once with PBS and incubated in assay medium at 37 °C with 5% CO_2_ overnight (16~24 h). The cells were then harvested, rinsed twice with cold staining buffer, and incubated with 1.0 μg/mL mouse anti-human GLP-2R monoclonal antibody or mouse IgG isotype control on ice for 30~45 min in the dark. After staining, the cells were washed twice with staining buffer and then resuspended in the same buffer for analysis. All of the data were acquired using an Attune NxT Flow Cytometer (Thermo Fisher Scientific, Waltham, MA, USA).

### 4.6. Reporter Gene Assay Procedure

Cell-based RGA has been described previously [41]. Briefly, 50 microliters of HEK293-GLP-2R-Luc cells were seeded into a 96-well white plate at a density of 4 × 10^4^ cells/well. The cells were then incubated at 37 °C with 5% CO_2_ overnight (16 to 24 h). The next day, the teduglutide was serially diluted 7-fold in culture medium at a starting concentration of 10 µg/mL, corresponding to 2.665 µmol/L. Fifty microliters of diluted teduglutide were then added to each well. Notably, the final concentrations of teduglutide ranged from 5000 ng/mL to 0.000124 ng/mL. After 5 h of incubation, 100 µL of britelite plus reporter Gene Assay reagent were added to each well and mixed thoroughly under subdued light conditions for 5 min. The RLU signal was then determined with a SpectraMax M5e multimode plate reader (Molecular Devices, San Jose, CA, USA).

### 4.7. Validation of the RGA

The RGA was validated as described previously [41]. Briefly, the teduglutide standard reference was diluted in the culture medium to five starting concentrations, which were 5, 7.1, 10, 14.1, and 20 µg/mL. Another independently prepared 10 µg/mL teduglutide standard reference sample was used as the in-house reference. The expected relative potencies of the teduglutide dilutions were 50%, 71%, 100%, 141%, and 200%. Samples of each potency level were tested by two analysts 8 times in total. To investigate whether HEK293-GLP-2R-Luc cells also respond to GIP, GLP-1, rCG, rLH, IL-2, IL-4, and IL-6, all those samples were diluted to 2.665 µmol/L and further diluted 7-fold for 10 concentration points. The response of HEK293-GLP2R-Luc cells to these samples were determined as illustrated in 4.6.

### 4.8. Stability Testing of Teduglutide with the RGA

Teduglutide DS was kept at 37 °C for 1, 3, 5, 8,12, and 14 days, while the non-treated DS was preserved at −80 °C. The bioactivities of the treated and non-treated DS were then measured by the RGA. The teduglutide contents were analyzed by HPLC. An XBridge Peptide BEH C18 column (300 A, 5 μm, 4.6 mm × 250 mm) purchased from Waters (Milford, MA, USA) was used with a flow rate of 1.0 mL/min and was detected at 214 nm. The mobile phase was aqueous solution containing 0.1% trifluoroacetic acid and acetonitrile containing 0.1% trifluoroacetic acid. A total of 50 μL DS samples were injected into the column. The relative content of teduglutide was determined by teduglutide peak area ratio of DS subjected to a temperature of 37 °C to the non-treated DS.

### 4.9. RNA Sequencing (RNA-seq)

For the preparation of total RNA for RNA-seq, HEK293-GLP-2R-Luc cells cultured in 6-well plate were treated with 10 µg/mL teduglutide, apraglutide, glepaglutide, elsiglutide, or DMSO for 15 h or 24 h. Total RNA was extracted with RNeasy mini kit (Qiagen, 74104, Hilden, Germany). A minimum of 2 μg of total RNA obtained from a mixture of 3-wells of cells were used for library construction. Library sequencing was performed on the Illumina HiSeq-PE150 platform (San Diego, CA, USA). RNA-seq raw data has been submitted to NCBI, and the accession numbers will be provided during review.

### 4.10. Statistical Analysis

For in vitro RGA, a 4PL model was used to fit the dose-response curve, which expresses the relationship between the RLU and log10 of the 10 points of teduglutide concentration. The lower/upper asymptote was determined by the ratio of the top asymptote to the bottom asymptote when RGA best fit the 4PL model. The correlation coefficient (R^2^), deviations from parallelism, and the regression in the 4PL model were estimated using SoftMax^®^ Pro 7.1 software (Molecular Devices, San Jose, CA, USA). Specifically, a linear regression with an R^2^ value no less than 0.98 was considered to have a very good fit. Additionally, a *p*-value below 0.01 was regarded as statistically significant. The relative potency of a sample was indicated by the EC50 ratio of the teduglutide standard reference to the teduglutide testing sample, resulting from the constraints of the 4PL model. All the dose-response curves were plotted using GraphPad Prism^®^ software 8.0.2 (GraphPad, Boston, MA, USA).

## 5. Conclusions

In this study, we successfully developed a robust and reproducible RGA utilizing a stable HEK293 cell line expressing the GLP-2 receptor and a luciferase reporter gene. The RGA demonstrated acceptable relative bias, intermediate precision, and linearity within the tested range. Through validation, the specificity and stability of the RGA were confirmed. Additionally, the RGA was capable of monitoring the reduction of teduglutide bioactivity. Our findings provide a sensitive and specific system for detecting GLP-2-mediated biological activities, offering valuable insights into the potential mechanisms of action of GLP-2 analogues.

## 6. Patents

A Chinese patent titled “A cell for determining the biological activity of GLP-2 analogues” (ZL202411308318.6) has been granted by the China National Intellectual Property Administration based on this research.

## Figures and Tables

**Table 1:** **Validation of relative accuracy, linearity, and precision of the RGA.** CI: confidence interval; GCV: geometric coefficient of variation.

Expected bioactivity	50%		71%		100%		141%		200%	
Round	A	B	A	B	A	B	A	B	A	B
1	0.518	0.532	0.763	0.653	1.048	0.970	1.504	1.482	1.879	2.091
2	0.502	0.524	0.772	0.660	1.069	0.961	1.496	1.446	2.145	2.252
3	0.541	0.532	0.727	0.677	0.902	1.116	1.484	1.387	1.994	2.415
4	0.465	0.530	0.702	0.734	0.988	1.004	1.294	1.532	2.373	1.969
Mean potency	0.518		0.711		1.007		1.453		2.140	
RSD of mean potency	4.695%		6.368%		6.763%		5.327%		9.069%	
90% CI of mean potency	(0.502, 0.534)		(0.681, 0.741)		(0.962, 1.053)		(1.401, 1.505)		(2.010, 2.269)	
Mean Ln potency	−0.659		−0.343		0.005		0.372		0.757	
90% CI of mean Ln potency	(−0.691, −0.626)		(−0.385, −0.300)		(−0.040, 0.050)		(0.336, 0.409)		(0.697, 0.817)	
Mean relative bias	3.512		−0.034		0.532		2.923		6.599	
90% CI of relative bias	(0.210, 6.923)		(−4.204, 4.318)		(−3.908, 5.176)		(−0.790, 6.775)		(0.351, 13.236)	
% GCV	4.971		6.584		6.993		5.652		9.460	

**Table 2:** Amino acid sequence and molecular weight of GLP-2 analogues.

Peptide	Mol. wt	Amino Acid Sequence
Teduglutide	3752.1	His-Gly-Asp-Gly-Ser-Phe-Ser-Asp-Glu-Met-Asn-Thr-Ile-Leu-Asp-Asn-Leu-Ala-Ala-Arg-Asp-Phe-Ile-Asn-Trp-Leu-Ile-Gln-Thr-Lys-Ile-Thr-Asp
Apraglutide	3765.0	His-Gly-Asp-Gly-Ser-Phe-Ser-Asp-Glu-Nle-{D-Phe}-Thr-Ile-Leu-Asp-Leu-Leu-Ala-Ala-Arg-Asp-Phe-Ile-Asn-Trp-Leu-Ile-Gln-Thr-Lys-Ile-Thr-Asp-NH2
Glepaglutide	4358.5	His-Gly-Glu-Gly-Thr-Phe-Ser-Ser-Glu-Leu-Ala-Thr-Ile-Leu-Asp-Ala-Leu-Ala-Ala-Arg-Asp-Phe-Ile-Asn-Trp-Leu-Ile-Ala-Thr-Lys-Ile-Thr-Asp-Lys-Lys-Lys-Lys-Lys-Lys-NH2
Elsiglutide	4362.0	His-Gly-Glu-Gly-Ser-Phe-Ser-Ser-Glu-Leu-Ser-Thr-Ile-Leu-Asp-Ala-Leu-Ala-Ala-Arg-Asp-Phe-Ile-Asn-Trp-Leu-Ile-Ala-Thr-Lys-Ile-Thr-Asp-Lys-Lys-Lys-Lys-Lys-Lys

## Data Availability

The data presented in this study are available upon request from the corresponding author. The data are not publicly available due to legal issues.

## Appendix A

Figure A1 shows a schematic of the GLP2-PKA-CREB signaling pathway. Table A1 lists the genes that are differentially expressed by more than 2-fold in HEK293-GLP-2R-Luc cells treated with apraglutide, glepaglutide, or elsiglutide, compared to those treated with DMSO. Table A2, on the other hand, lists the genes that are differentially expressed by more than 2-fold exclusively in HEK293-GLP-2R-Luc cells treated with elsiglutide.

**Table A1:** Differentially expressed genes in GLP-2 analogues apraglutide, glepaglutide, or elsiglutide treated HEK293-GLP-2R-Luc cells.

Gene ID	Gene Symbol	log2 (TD/Ctrl)	Qvalue × 10 (TD/Ctrl)
22987	‘SV2C’	6.783231226	2.52 × 10^−39^
1081	‘CGA’	6.562903052	3.88 × 10^−72^
5122	‘PCSK1’	4.991509306	2.68 × 10^−35^
56892	‘TCIM’	4.105467778	2.79 × 10^−4^
2118	‘ETV4’	3.754600881	6.41 × 10^−55^
4922	‘NTS’	3.486124464	5.17 × 10^−6^
133688	‘UGT3A1’	3.442316651	7.81 × 10^−4^
92737	‘DNER’	3.335646375	3.18 × 10^−21^
1114	‘CHGB’	3.025006026	4.61 × 10^−15^
3624	‘INHBA’	2.908922408	5.76 × 10^−13^
3164	‘NR4A1’	2.867922186	0.017911889
10083	‘USH1C’	2.824321935	1.03 × 10^−8^
168667	‘BMPER’	2.760986666	1.19 × 10^−7^
6863	‘TAC1’	2.758702523	6.30 × 10^−16^
6860	‘SYT4’	2.681770567	5.46 × 10^−4^
5139	‘PDE3A’	2.655556063	5.57 × 10^−6^
1600	‘DAB1’	2.621837125	3.67 × 10^-8^
5105	‘PCK1’	2.587238646	0.040888626
4929	‘NR4A2’	2.397468553	3.82 × 10^−6^
7425	‘VGF’	2.119321114	3.44 × 10^−6^
134111	‘UBE2QL1’	2.107740434	1.60 × 10^−14^
92949	‘ADAMTSL1’	2.10209024	0.001469034
797	‘CALCB’	2.074574214	2.08 × 10^−12^
2119	‘ETV5’	2.053615447	1.70 × 10^−22^
2827	‘GPR3’	1.963250916	0.002106675
8061	‘FOSL1’	1.928539398	3.20 × 10^−5^
23764	‘MAFF’	1.865094918	0.001121908
286133	‘SCARA5’	1.827624849	0.017122186
27115	‘PDE7B’	1.707402315	0.036389414
8013	‘NR4A3’	1.676175444	4.33 × 10^−8^
5069	‘PAPPA’	1.666266407	1.03 × 10^−4^
387763	‘C11orf96’	1.665883142	3.60 × 10^−9^
1848	‘DUSP6’	1.616560238	2.50 × 10^−5^
93953	‘GCNA’	1.615861899	1.19 × 10^−18^
81848	‘SPRY4’	1.60022244	8.32 × 10^−5^
6616	‘SNAP25’	1.562181737	1.28 × 10^−4^
5142	‘PDE4B’	1.540946952	1.40 × 10^−4^
1390	‘CREM’	1.511462243	2.22 × 10^−28^
5166	‘PDK4’	1.496072406	2.85 × 10^−8^
5475	‘PPEF1’	1.488927063	0.009480869
1277	‘COL1A1’	1.488533782	8.10 × 10^−12^
146206	‘CARMIL2’	1.478259398	1.29 × 10^−6^
388662	‘SLC6A17’	1.470967211	0.002568883
102724428	‘LOC102724428’	1.465317035	4.53 × 10^−10^
1846	‘DUSP4’	1.423943562	4.96 × 10^−6^
8870	‘IER3’	1.412592552	4.26 × 10^−9^
55603	‘TENT5A’	1.410930069	1.48 × 10^−17^
94056	‘SYAP1’	1.386934782	2.87 × 10^−50^
3642	‘INSM1’	1.381342648	0.029618286
54498	‘SMOX’	1.374084566	1.43 × 10^−7^
9427	‘ECEL1’	1.362369453	0.021513752
10846	‘PDE10A’	1.350405481	1.23 × 10^−22^
150094	‘SIK1’	1.314138132	0.046326139
3371	‘TNC’	1.311814046	2.88 × 10^−32^
9388	‘LIPG’	1.309329576	2.43 × 10^−9^
6446	‘SGK1’	1.306069633	1.32 × 10^−7^
3576	‘CXCL8’	1.295326935	0.029142572
56475	‘RPRM’	1.288867868	3.11 × 10^−18^
100133941	‘CD24’	1.239458742	1.01 × 10^−9^
2717	‘GLA’	1.223468069	5.79 × 10^−21^
84034	‘EMILIN2’	1.218499891	1.56 × 10^−12^
1490	‘CCN2’	1.202513938	0.010818778
196	‘AHR’	1.18672228	2.72 × 10^−13^
2669	‘GEM’	1.14872712	6.89 × 10^−4^
5270	‘SERPINE2’	1.143281421	1.81 × 10^−11^
1604	‘CD55’	1.135266565	9.57 × 10^−21^
9172	‘MYOM2’	1.13064119	1.55 × 10^−4^
131583	‘FAM43A’	1.108660396	0.004172994
89797	‘NAV2’	1.098948271	5.93 × 10^−7^
4330	‘MN1’	1.082766426	0.027845365
7803	‘PTP4A1’	1.072165321	3.56 × 10^−31^
10397	‘NDRG1’	1.067453358	0.005973099
1843	‘DUSP1’	1.044054019	4.64 × 10^−12^
783	‘CACNB2’	1.036050346	0.02386605
5144	‘PDE4D’	1.02966789	7.07 × 10^−11^
1831	‘TSC22D3’	1.013141919	9.09 × 10^−16^
124901228	‘LOC124901228’	−1.034748741	0.036704524
64084	‘CLSTN2’	−1.06332157	0.013853614
260293	‘CYP4 × 1’	−1.143974821	1.60 × 10^−7^
59	‘ACTA2’	−1.17154371	1.33 × 10^−4^
3162	‘HMOX1’	−1.206862228	4.89 × 10^−8^
3635	‘INPP5D’	−2.361870877	4.09 × 10^−5^
8681	‘JMJD7-PLA2G4B’	−3.917402548	0.012575826

**Table A2:** Differentially expressed genes in glepaglutide-treated HEK293-GLP-2R-Luc cells.

Gene ID	Gene Symbol	log2 (TD/Ctrl)	Qvalue (TD/Ctrl)
1081	‘CGA’	7.041949	7.84 × 10^−76^
56892	‘TCIM’	7.036603	2.51 × 10^−19^
22987	‘SV2C’	6.845154	1.91 × 10^−39^
1.01 × 10^8^	‘FAM47E-STBD1’	6.132326	0.014365
51200	‘CPA4’	5.505632	2.40 × 10^−4^
5122	‘PCSK1’	5.002027	5.37 × 10^−34^
5105	‘PCK1’	4.953544	5.99 × 10^−8^
6863	‘TAC1’	4.688664	4.80 × 10^−76^
3624	‘INHBA’	4.677791	9.22 × 10^−49^
9628	‘RGS6’	4.489375	8.61 × 10^−5^
387763	‘C11orf96’	4.460874	3.88 × 10^−22^
3164	‘NR4A1’	4.425716	8.67 × 10^−7^
4922	‘NTS’	3.900682	4.98 × 10^−8^
50805	‘IRX4’	3.763052	1.00 × 10^−4^
3872	‘KRT17’	3.685233	7.46 × 10^−4^
797	‘CALCB’	3.65835	4.28 × 10^−58^
2118	‘ETV4’	3.646442	2.09 × 10^−50^
23764	‘MAFF’	3.638742	2.58 × 10^−27^
80117	‘ARL14’	3.565302	2.71 × 10^−4^
374	‘AREG’	3.50869	6.48 × 10^−5^
1844	‘DUSP2’	3.424234	0.008669
1114	‘CHGB’	3.305006	5.45 × 10^−20^
286133	‘SCARA5’	3.28416	4.60 × 10^−12^
2353	‘FOS’	3.219529	1.54 × 10^−5^
8061	‘FOSL1’	3.192743	5.84 × 10^−23^
133688	‘UGT3A1’	3.069411	0.004706
5798	‘PTPRN’	3.046171	0.005327
92737	‘DNER’	3.017994	1.71 × 10^−14^
2827	‘GPR3’	3.012651	1.05 × 10^−12^
7425	‘VGF’	2.990033	1.36 × 10^−15^
1958	‘EGR1’	2.978485	6.76 × 10^−10^
27063	‘ANKRD1’	2.953204	0.012462
9047	‘SH2D2A’	2.921605	0.005953
2669	‘GEM’	2.896159	1.30 × 10^−33^
388	‘RHOB’	2.852415	0.003482
1843	‘DUSP1’	2.837365	1.23 × 10^−13^
2354	‘FOSB’	2.796243	1.44 × 10^−4^
388662	‘SLC6A17’	2.783714	2.12 × 10^−19^
1306	‘COL15A1’	2.733418	0.016322
1.28 × 10^8^	‘LOC128125822’	2.711096	0.008527
345651	‘ACTBL2’	2.679934	1.10 × 10^−21^
4319	‘MMP10’	2.629886	4.91 × 10^−4^
219539	‘YPEL4’	2.620859	2.87 × 10^−4^
1761	‘DMRT1’	2.577685	0.038243
1490	‘CCN2’	2.557624	5.36 × 10^−15^
23237	‘ARC’	2.54751	4.77 × 10^−21^
10083	‘USH1C’	2.546777	3.44 × 10^−6^
3589	‘IL11’	2.542745	0.014439
467	‘ATF3’	2.526935	3.77 × 10^−12^
54742	‘LY6K’	2.444329	5.54 × 10^−6^
4929	‘NR4A2’	2.427716	7.07 × 10^−23^
6303	‘SAT1’	2.41562	0.002832
84665	‘MYPN’	2.401189	0.005296
3575	‘IL7R’	2.399983	0.003482
55790	‘CSGALNACT1’	2.392056	3.63 × 10^−4^
7980	‘TFPI2’	2.377004	2.01 × 10^−13^
10170	‘DHRS9’	2.367053	0.007207
6860	‘SYT4’	2.338338	0.005991
1 × 10^8^	‘CD24’	2.289223	8.27 × 10^−27^
9715	‘FAM131B’	2.283871	4.57 × 10^−4^
51655	‘RASD1’	2.272767	3.31 × 10^−6^
3576	‘CXCL8’	2.264138	4.89 × 10^−10^
55328	‘RNLS’	2.250637	0.001684
10044	‘SH2D3C’	2.238657	4.00 × 10^−6^
1960	‘EGR3’	2.228172	9.86 × 10^−8^
84069	‘PLEKHN1’	2.209516	0.012462
2151	‘F2RL2’	2.195009	0.035979
3726	‘JUNB’	2.192255	2.71 × 10^−23^
51330	‘TNFRSF12A’	2.18285	2.59 × 10^−23^
432	‘ASGR1’	2.135498	0.00679
168667	‘BMPER’	2.129836	8.87 × 10^−4^
960	‘CD44’	2.114616	1.93 × 10^−23^
170261	‘ZCCHC12’	2.109762	6.60 × 10^−42^
9119	‘KRT75’	2.098589	0.012583
10215	‘OLIG2’	2.054422	2.31 × 10^−9^
64651	‘CSRNP1’	2.050418	1.62 × 10^−22^
22941	‘SHANK2’	2.034944	0.007984
9427	‘ECEL1’	2.017527	1.32 × 10^−6^
968	‘CD68’	2.011516	2.06 × 10^−17^
114801	‘TMEM200A’	2.008387	0.038553
9248	‘GPR50’	2.001869	2.38 × 10^−8^
4616	‘GADD45B’	1.994977	9.33 × 10^−42^
2675	‘GFRA2’	1.989401	0.036382
79845	‘RNF122’	1.985006	5.63 × 10^−24^
57596	‘BEGAIN’	1.974158	3.50 × 10^−4^
5971	‘RELB’	1.9665	1.72 × 10^−7^
26353	‘HSPB8’	1.961138	5.44 × 10^−4^
5260	‘PHKG1’	1.954624	0.021994
1.01 × 10^8^	‘MICOS10-NBL1’	1.951595	0.023249
200407	‘CREG2’	1.949279	0.012071
2119	‘ETV5’	1.931992	5.77 × 10^−17^
10365	‘KLF2’	1.927508	0.005739
1846	‘DUSP4’	1.926226	2.57 × 10^−12^
1959	‘EGR2’	1.924048	0.027245
1277	‘COL1A1’	1.910436	8.07 × 10^−20^
3604	‘TNFRSF9’	1.903185	3.27 × 10^−6^
134111	‘UBE2QL1’	1.897385	5.89 × 10^−10^
5139	‘PDE3A’	1.882402	0.023634
9846	‘GAB2’	1.866086	6.27 × 10^−10^
23349	‘PHF24’	1.838749	0.018911
3491	‘CCN1’	1.829785	1.09 × 10^−13^
8013	‘NR4A3’	1.795425	6.44 × 10^−8^
7262	‘PHLDA2’	1.790142	3.51 × 10^−4^
387755	‘INSC’	1.775005	0.033388
6578	‘SLCO2A1’	1.773488	0.001637
92949	‘ADAMTSL1’	1.769459	0.016921
146206	‘CARMIL2’	1.753367	7.13 × 10^−10^
7087	‘ICAM5’	1.744337	1.87 × 10^−4^
3570	‘IL6R’	1.741761	2.27 × 10^−19^
93145	‘OLFM2’	1.731761	3.15 × 10^−7^
23645	‘PPP1R15A’	1.729441	4.52 × 10^−19^
602	‘BCL3’	1.727758	0.020272
112885	‘PHF21B’	1.711241	3.56 × 10^−4^
6616	‘SNAP25’	1.697534	4.24 × 10^−5^
4135	‘MAP6’	1.691121	0.009857
7040	‘TGFB1’	1.682914	1.04 × 10^−9^
2717	‘GLA’	1.679692	2.98 × 10^−32^
3601	‘IL15RA’	1.677225	3.17 × 10^−4^
8870	‘IER3’	1.67673	1.44 × 10^−12^
7538	‘ZFP36’	1.675899	2.68 × 10^−12^
10397	‘NDRG1’	1.674325	1.40 × 10^−27^
1831	‘TSC22D3’	1.669903	3.35 × 10^−6^
342865	‘VSTM2B’	1.667679	0.024619
729440	‘CCDC61’	1.662713	0.003634
3371	‘TNC’	1.65734	2.37 × 10^−34^
90874	‘ZNF697’	1.651038	6.15 × 10^−17^
7277	‘TUBA4A’	1.633093	2.12 × 10^−4^
3592	‘IL12A’	1.606249	5.36 × 10^−4^
93953	‘GCNA’	1.602272	4.80 × 10^−17^
666	‘BOK’	1.598824	0.002402
81848	‘SPRY4’	1.582533	1.22 × 10^−4^
5069	‘PAPPA’	1.57807	5.75 × 10^−4^
1647	‘GADD45A’	1.572569	1.52 × 10^−19^
7975	‘MAFK’	1.560582	1.26 × 10^−13^
5329	‘PLAUR’	1.559275	6.86 × 10^−10^
1604	‘CD55’	1.554485	4.43 × 10^−29^
4791	‘NFKB2’	1.551281	9.77 × 10^−25^
25791	‘NGEF’	1.548573	0.001858
5475	‘PPEF1’	1.542688	0.002369
1600	‘DAB1’	1.527003	0.044461
84667	‘HES7’	1.524518	0.024442
3761	‘KCNJ4’	1.520604	0.028153
2012	‘EMP1’	1.518486	0.001666
153769	‘SH3RF2’	1.51636	0.008648
94056	‘SYAP1’	1.501243	6.03 × 10^−37^
7803	‘PTP4A1’	1.500196	1.47 × 10^−41^
27087	‘B3GAT1’	1.484664	3.15 × 10^−7^
54498	‘SMOX’	1.478782	1.04 × 10^−10^
26038	‘CHD5’	1.476466	0.036855
5166	‘PDK4’	1.463191	4.05 × 10^−7^
4915	‘NTRK2’	1.454183	0.006147
29950	‘SERTAD1’	1.451638	2.03 × 10^−14^
1 × 10^8^	‘TMEM225B’	1.447921	0.010366
752	‘FMNL1’	1.446434	0.018333
842	‘CASP9’	1.439331	3.78 × 10^−13^
25780	‘RASGRP3’	1.425614	0.017723
23710	‘GABARAPL1’	1.405278	8.03 × 10^−15^
1316	‘KLF6’	1.401921	0.022289
7280	‘TUBB2A’	1.401486	2.02 × 10^−19^
5272	‘SERPINB9’	1.400471	9.32 × 10^−11^
1263	‘PLK3’	1.39147	2.50 × 10^−4^
81606	‘LBH’	1.386926	1.62 × 10^−11^
2355	‘FOSL2’	1.384896	1.49 × 10^−13^
1.03 × 10^8^	‘LOC102724428’	1.373016	1.72 × 10^−7^
4330	‘MN1’	1.348195	3.74 × 10^−4^
5997	‘RGS2’	1.337363	3.01 × 10^−9^
23308	‘ICOSLG’	1.336938	0.015122
9052	‘GPRC5A’	1.32849	2.28 × 10^−8^
3914	‘LAMB3’	1.322703	9.88 × 10^−5^
6446	‘SGK1’	1.322445	1.39 × 10^−7^
138428	‘PTRH1’	1.321988	7.65 × 10^−4^
9510	‘ADAMTS1’	1.320096	6.10 × 10^−16^
1390	‘CREM’	1.319468	1.18 × 10^−18^
1491	‘CTH’	1.317447	5.77 × 10^−13^
25822	‘DNAJB5’	1.315728	1.56 × 10^−4^
23105	‘FSTL4’	1.312165	0.011445
80328	‘ULBP2’	1.311597	3.54 × 10^−4^
8612	‘PLPP2’	1.309113	0.006233
6692	‘SPINT1’	1.306612	1.47 × 10^−8^
65009	‘NDRG4’	1.306216	8.22 × 10^−4^
84034	‘EMILIN2’	1.304201	1.41 × 10^−13^
1969	‘EPHA2’	1.302845	2.56 × 10^−11^
160428	‘ALDH1L2’	1.302838	0.012744
7378	‘UPP1’	1.295699	2.29 × 10^−9^
55647	‘RAB20’	1.264923	0.026593
8646	‘CHRD’	1.26076	0.010428
3642	‘INSM1’	1.258868	0.02493
8349	‘H2BC21’	1.258639	0.004109
150094	‘SIK1’	1.255484	0.025061
24142	‘NAA80’	1.249398	0.008009
9902	‘MRC2’	1.244459	4.00 × 10^−5^
2318	‘FLNC’	1.242928	2.88 × 10^−7^
91107	‘TRIM47’	1.231131	2.06 × 10^−4^
7128	‘TNFAIP3’	1.226778	1.80 × 10^−7^
389792	‘IER5L’	1.226594	0.037055
255057	‘CBARP’	1.225496	7.76 × 10^−5^
22822	‘PHLDA1’	1.22424	1.40 × 10^−4^
90139	‘TSPAN18’	1.221107	6.12 × 10^−5^
3725	‘JUN’	1.215714	3.59 × 10^−15^
330	‘BIRC3’	1.21349	0.038759
80758	‘PRR7’	1.208902	0.002734
10544	‘PROCR’	1.200729	5.32 × 10^−8^
7409	‘VAV1’	1.197633	3.01 × 10^−4^
84879	‘MFSD2A’	1.192823	1.88 × 10^−5^
22936	‘ELL2’	1.192026	1.93 × 10^−15^
60370	‘AVPI1’	1.191713	5.33 × 10^−8^
7781	‘SLC30A3’	1.181676	0.007467
4783	‘NFIL3’	1.176892	2.91 × 10^−8^
5292	‘PIM1’	1.176599	3.61 × 10^−6^
120071	‘LARGE2’	1.169959	0.023651
9592	‘IER2’	1.167132	9.14 × 10^−13^
54795	‘TRPM4’	1.16361	0.034028
11151	‘CORO1A’	1.157197	0.03809
1303	‘COL12A1’	1.157193	3.45 × 10^−9^
9625	‘AATK’	1.156459	0.001714
10045	‘SH2D3A’	1.146406	0.013825
4772	‘NFATC1’	1.146342	1.32 × 10^−9^
653333	‘FAM86B2’	1.144232	4.40 × 10^−6^
54033	‘RBM11’	1.142746	0.001608
79094	‘CHAC1’	1.140785	4.05 × 10^−4^
26093	‘CCDC9’	1.137033	2.50 × 10^−4^
5270	‘SERPINE2’	1.135561	7.93 × 10^−10^
999	‘CDH1’	1.134996	0.049404
53405	‘CLIC5’	1.134372	0.046355
79603	‘CERS4’	1.130644	0.01208
79924	‘ADM2’	1.126908	0.018318
79583	‘TMEM231’	1.1218	3.45 × 10^−5^
129293	‘TRABD2A’	1.121194	0.026368
9201	‘DCLK1’	1.118285	4.77 × 10^−6^
114789	‘SLC25A25’	1.116863	2.28 × 10^−9^
64399	‘HHIP’	1.113503	0.016047
8038	‘ADAM12’	1.111516	0.001549
132864	‘CPEB2’	1.107394	1.61 × 10^−6^
2786	‘GNG4’	1.107044	1.34 × 10^−10^
7035	‘TFPI’	1.10253	1.19 × 10^−6^
89797	‘NAV2’	1.101959	2.21 × 10^−5^
6591	‘SNAI2’	1.100409	0.046941
24139	‘EML2’	1.097709	1.31 × 10^−5^
65117	‘RSRC2’	1.090294	5.28 × 10^−10^
89849	‘ATG16L2’	1.087424	0.006489
7171	‘TPM4’	1.085642	1.41 × 10^−16^
7942	‘TFEB’	1.085071	0.014032
64285	‘RHBDF1’	1.083098	0.005404
4804	‘NGFR’	1.082959	0.0305
2274	‘FHL2’	1.081722	9.62 × 10^−6^
9945	‘GFPT2’	1.080437	1.35 × 10^−4^
8622	‘PDE8B’	1.080217	4.35 × 10^−6^
782	‘CACNB1’	1.074917	4.04 × 10^−5^
113452	‘TMEM54’	1.072456	0.021231
348093	‘RBPMS2’	1.068707	0.001565
654364	‘NME1-NME2’	1.068581	6.18 × 10^−4^
84803	‘GPAT3’	1.067199	1.61 × 10^−6^
9961	‘MVP’	1.066078	0.048413
27289	‘RND1’	1.057224	0.008622
6261	‘RYR1’	1.054767	0.021465
65249	‘ZSWIM4’	1.053674	0.001797
400745	‘SH2D5’	1.053388	0.00738
283991	‘UBALD2’	1.052934	2.76 × 10^−6^
7461	‘CLIP2’	1.05197	0.008212
10846	‘PDE10A’	1.051532	4.45 × 10^−11^
1545	‘CYP1B1’	1.051511	8.07 × 10^−4^
22888	‘UBOX5’	1.051382	0.00141
57132	‘CHMP1B’	1.047506	6.69 × 10^−14^
306	‘ANXA3’	1.046248	0.034821
2275	‘FHL3’	1.044795	3.94 × 10^−5^
55422	‘ZNF331’	1.04065	8.43 × 10^−12^
57602	‘USP36’	1.03853	1.63 × 10^−11^
84446	‘BRSK1’	1.032849	4.84 × 10^−4^
440829	‘SHISA8’	1.031009	0.048554
91862	‘MARVELD3’	1.030794	0.011267
2979	‘GUCA1B’	1.025814	0.013456
100	‘ADA’	1.022892	1.89 × 10^−4^
5366	‘PMAIP1’	1.019573	1.07 × 10^−13^
135154	‘SDHAF4’	1.017915	0.002952
196	‘AHR’	1.016113	1.47 × 10^−8^
84210	‘ANKRD20A1’	1.015458	0.047154
148113	‘CILP2’	1.013671	0.028089
1.01 × 10^8^	‘MSANTD3-TMEFF1’	1.009247	3.37 × 10^−6^
60487	‘TRMT11’	1.007178	4.83 × 10^−7^
162979	‘ZNF296’	1.004574	0.049596
7375	‘USP4’	1.00177	1.38 × 10^−4^
159091	‘PABIR3’	1.00145	0.044085
79101	‘TAF1D’	1.000969	6.18 × 10^−6^
64921	‘CASD1’	−1.00687	1.39 × 10^−4^
79685	‘SAP30L’	−1.0123	9.88 × 10^−7^
7582	‘ZNF33B’	−1.02141	2.82 × 10^−4^
29071	‘C1GALT1C1’	−1.02185	2.74 × 10^−4^
64789	‘EXO5’	−1.02321	0.020878
4091	‘SMAD6’	−1.02343	1.54 × 10^−5^
7442	‘TRPV1’	−1.03801	0.001125
2342	‘FNTB’	−1.03935	0.011191
84190	‘METTL25’	−1.04498	0.001214
124936	‘CYB5D2’	−1.05624	1.97 × 10^−6^
284086	‘NEK8’	−1.05725	0.001147
493911	‘PHOSPHO2’	−1.05875	0.023229
9901	‘SRGAP3’	−1.06252	0.002313
642574	‘IQANK1’	−1.06993	0.02555
8322	‘FZD4’	−1.07057	8.54 × 10^−5^
9758	‘FRMPD4’	−1.07796	2.07 × 10^−4^
5087	‘PBX1’	−1.09531	1.77 × 10^−11^
8516	‘ITGA8’	−1.10169	0.001396
353355	‘ZNF233’	−1.10239	0.018109
63939	‘FAM217B’	−1.10601	2.42 × 10^−6^
2982	‘GUCY1A1’	−1.10666	4.19 × 10^−5^
2650	‘GCNT1’	−1.11342	1.22 × 10^−6^
57545	‘CC2D2A’	−1.1174	0.001438
268	‘AMH’	−1.12163	0.001638
84078	‘KBTBD7’	−1.12202	6.73 × 10^−6^
54863	‘TOR4A’	−1.12273	0.034573
130574	‘LYPD6’	−1.12436	1.04 × 10^−4^
581	‘BAX’	−1.136	1.10 × 10^−10^
9415	‘FADS2’	−1.13601	1.54 × 10^−10^
4133	‘MAP2’	−1.13764	0.019619
55924	‘INKA2’	−1.14012	5.19 × 10^−4^
5101	‘PCDH9’	−1.15797	5.30 × 10^−8^
728239	‘MAGED4’	−1.15943	0.009809
115209	‘OMA1’	−1.16485	6.34 × 10^−6^
90427	‘BMF’	−1.17844	1.77 × 10^−4^
5099	‘PCDH7’	−1.18497	3.52 × 10^−9^
6657	‘SOX2’	−1.18867	2.21 × 10^−5^
63920	‘FAM200C’	−1.19013	6.74 × 10^−4^
8829	‘NRP1’	−1.19113	2.55 × 10^−11^
285440	‘CYP4V2’	−1.19791	8.29 × 10^−5^
29923	‘HILPDA’	−1.21543	0.00213
55779	‘CFAP44’	−1.2179	1.21 × 10^−4^
374393	‘FAM111B’	−1.22249	9.51 × 10^−4^
91319	‘DERL3’	−1.23668	0.001478
1.25 × 10^8^	‘LOC124904248’	−1.23949	0.046402
57727	‘NCOA5’	−1.2433	5.31 × 10^−8^
84074	‘QRICH2’	−1.25334	8.25 × 10^−6^
3216	‘HOXB6’	−1.27389	1.35 × 10^−4^
115330	‘GPR146’	−1.28358	0.031918
80059	‘LRRTM4’	−1.28521	0.022315
84858	‘ZNF503’	−1.2971	1.87 × 10^−11^
1.25 × 10^8^	‘LOC124901228’	−1.29899	0.002022
255403	‘ZNF718’	−1.3027	6.33 × 10^−4^
1.01 × 10^8^	‘POC1B-GALNT4’	−1.31941	0.011547
389813	‘AJM1’	−1.32719	0.017304
7474	‘WNT5A’	−1.33174	2.87 × 10^−7^
9315	‘NREP’	−1.33909	3.01 × 10^−13^
5608	‘MAP2K6’	−1.34833	6.95 × 10^−9^
353497	‘POLN’	−1.36413	0.020952
60401	‘EDA2R’	−1.37352	9.85 × 10^−6^
387640	‘SKIDA1’	−1.40715	3.39 × 10^−5^
390637	‘GDPGP1’	−1.43035	0.011565
8793	‘TNFRSF10D’	−1.43223	5.55 × 10^−11^
2045	‘EPHA7’	−1.44304	6.41 × 10^−18^
79081	‘LBHD1’	−1.45339	1.34 × 10^−6^
80823	‘GPRASP3’	−1.47428	0.002224
221527	‘ZBTB12’	−1.60004	7.78 × 10^−9^
1294	‘COL7A1’	−1.61245	7.17 × 10^−4^
154796	‘AMOT’	−1.62021	2.58 × 10^−33^
101	‘ADAM8’	−1.63217	0.026923
79930	‘DOK3’	−1.63615	2.01 × 10^−5^
375519	‘GJB7’	−1.70619	0.040011
2298	‘FOXD4’	−1.76495	0.046871
284739	‘C20orf204’	−1.77711	0.021231
6335	‘SCN9A’	−1.80043	1.83 × 10^−18^
3488	‘IGFBP5’	−1.80308	4.48 × 10^−29^
56606	‘SLC2A9’	−1.82546	0.032594
84517	‘ACTRT3’	−1.88983	0.027976
10628	‘TXNIP’	−1.9141	2.97 × 10^−31^
387758	‘FIBIN’	−1.93086	0.032833
260293	‘CYP4 × 1’	−1.96314	1.72 × 10^−18^
414060	‘TBC1D3C’	−2.32118	0.04891
3635	‘INPP5D’	−2.41744	1.84 × 10^−5^
6387	‘CXCL12’	−2.42221	0.004272
6326	‘SCN2A’	−2.49417	0.014161
257044	‘CATSPERE’	−2.49598	0.013837
5032	‘P2RY11’	−9.7959	7.12 × 10^−9^
